# Spatiotemporal Analysis of Online Purchase of HIV Self-testing Kits in China, 2015-2017: Longitudinal Observational Study

**DOI:** 10.2196/37922

**Published:** 2022-09-27

**Authors:** Yi Lv, Qiyu Zhu, Chengdong Xu, Guanbin Zhang, Yan Jiang, Mengjie Han, Cong Jin

**Affiliations:** 1 National Center for AIDS/STD Control and Prevention Chinese Center for Disease Control and Prevention Beijing China; 2 State Key Laboratory of Resources and Environmental Information System Institute of Geographic Sciences and Natural Resources Research Chinese Academy of Sciences Beijing China; 3 National Engineering Research Center for Beijing Biochip Technology Beijing China; 4 Department of Infectious Diseases 404 Hospital of Mianyang Sichuan China; 5 Chinese Association of STD and AIDS Prevention and Control Beijing China

**Keywords:** spatiotemporal, characteristics, online, purchase, HIV, self-testing, e-commerce, economic status, HIV epidemic, China

## Abstract

**Background:**

Since the introduction of HIV self-testing by UNAIDS in 2014, the practice has been extensively implemented around the world. HIV self-testing (HIVST) was developed in China around 2015, and the online purchase of HIVST kits through e-commerce platforms has since become the most important delivery method for self-testing, with advantages such as user-friendliness, speed, and better privacy protection.

**Objective:**

Understanding the spatiotemporal characteristics of online HIVST kit purchasing behavior and identifying potential impacting factors will help promote the HIV self-testing strategy.

**Methods:**

The online retail data of HIVST kits from the 2 largest e-commerce platforms in China from 2015 to 2017 were collected for this study. The Bayesian spatiotemporal hierarchical model was used to investigate the spatiotemporal characteristics of online purchased HIVST kits. Ordinary least squares regression was used to identify potential factors associated with online purchase, including GDP per capita, population density, road density, HIV screening laboratory density, and newly diagnosed HIV/AIDS cases per 100,000 persons. The *q* statistics calculated by Geodetector were used to determine the interactive effect of every 2 factors on the online purchase.

**Results:**

The online purchase of HIVST kits increased rapidly in China from 2015 to 2017, with annual peak sales in May and December. Five economically superior regions in China, Pearl River Delta, Yangtze River Delta, Chengdu and surrounding areas, Beijing and Tianjin areas, and Shandong Peninsula, showed a comparatively higher spatial preference for online purchased HIVST kits. The GDP per capita (*P*<.001) and the rate of newly diagnosed HIV/AIDS cases per 100,000 persons (*P*<.001) were identified as 2 factors positively associated with online purchase. Among the factors we investigated in this study, 2 factors associated with online purchase, GDP per capita and the rate of newly diagnosed HIV/AIDS cases per 100,000 persons, also displayed the strongest interactive effect, with a *q* value of 0.66.

**Conclusions:**

Individuals in better-off areas are more inclined to purchase HIVST kits online. In addition to economic status, the severity of the HIV epidemic is also a factor influencing the online purchase of HIVST kits.

## Introduction

To help end the AIDS epidemic, the Joint United Nations Program on HIV/AIDS (UNAIDS) proposed the 90-90-90 target in 2014 [1]. This ambitious but achievable target of 90% of people living with HIV being aware of their status [1] and that HIV testing services (HTS) play important roles and serve as the gateway to treatment, prevention, and care [2]. HIV self-testing (HIVST), defined as a type of testing strategy in which sample collection, testing, and interpretation are all performed by individuals who wish to learn about their HIV status on their own in a private environment, is known as a confidential way of HIV testing [3]. The interest in the use of HIV rapid diagnostic tests (RDTs) for self-testing has increased worldwide since 2015 [2]. As a private and convenient method, HIVST has been widely accepted by diverse populations, including those who might not otherwise be tested due to stigma and confidentiality concerns [4-6]. HIVST is especially cost-effective in resource-limited areas and has proven to be an effective way to expand HIV testing service [7]. As of June 2020, 41 countries around the world had implemented HIVST. In addition, 45 countries had allowed HIV self-testing policies [8].

The provider-initiated HIV testing and counseling (PITC) and voluntary counseling and testing (VCT) have been routinely provided in China. Since the World Health Organization advocated HIV self-testing in 2015 [2], a series of national policies on HIVST were implemented in China. Because HIVST kits belong to the third category of medical devices in China, under the regulations of the National Medical Products Administration, only a small number of pharmacies that had the business and sales qualification of the third category of medical devices were qualified to sell HIVST kits. The requirements of online HIVST retail are also very strict. To sell HIVST kits online, retailers need the business and sales qualification of the third category of medical devices and the business qualification of internet medical devices [9]. However, the logistics in China developed fast, and online retail could meet the purchasing needs of a wider area. Therefore, online purchase of HIVST kits through e-commerce platforms has become the most important form of delivery for private self-testing in China, with the advantages of being user-friendly, fast, and privacy protection. Purchasing HIVST kits online met the needs of certain populations who were reluctant to seek HIV testing services at health facilities. There are 3 types of HIVST kits sold online in China, including fingertip blood test kits, oral mucosal transudate test kits, and urine test kits [10]. The HIVST kits sold online range in price from 20 to 200 RMB (US $2.93-$29.34), with an average price of about 50 RMB (US $7.34) [11,12].

Since the introduction and promotion of HIVST in China, it has been gradually accepted and welcomed by users. It was reported that about 220 HIVST kits were sold per hour in 2017 by an online pharmaceutical store in China [13]. However, the characteristics of online HIVST purchasing behaviors have not been systematically studied. In this study, we analyzed the online HIVST kit sales data from 2015 to 2017 from JD and Taobao, the top 2 e-commerce platforms in China, to reveal the spatiotemporal characteristics of online HIVST kit purchases. Further, we investigated potential factors associated with online purchase of HIVST kits and roughly assessed the contribution of online acquisition of HIVST to HIV diagnosis. Our findings will inform the behavioral profile of those who purchase of HIVST kits online, which can further promote self-testing.

## Methods

### Data Source

JD and Taobao are two of the largest and most popular e-commerce platforms in China. The online retail data of HIVST kits at city level from 2015 to 2017 were collected from JD and Taobao, the largest retailer of HIVST kits, which sold 70% of HIVST kits. The online retail data we collected for this study had the city location of purchasers but did not include customer shipping addresses. City-level socioeconomic and demographic data were extracted from the Statistical Yearbook of each province or the official website of the local statistics department, including population, gross domestic product (GDP), and gross domestic product per capita (GDP per capita). Geographic data were downloaded from the National Catalogue Service for Geographic Information [14]. The data on newly diagnosed HIV cases at city level were collected from the provincial Centers for Disease Control and Prevention and the data center of China Public Health Science [15]. The data on HIV screening laboratories were obtained from the National HIV/AIDS Laboratory Management Information System of China.

### Spatiotemporal Distribution of the Online Purchase of HIVST Kits

The spatial and temporal characteristics of the online purchase of HIVST kits were investigated using the Bayesian spatiotemporal hierarchical model. To eliminate the influence of population size and better reflect the behavioral characteristics of people in the city purchasing HIVST kits online, the purchase rate of HIVST kits per capita was calculated by dividing the amount of HIVST kits sold in a city by the number of people living in that city. Poisson and log link regression functions were used to perform analyses as follows:



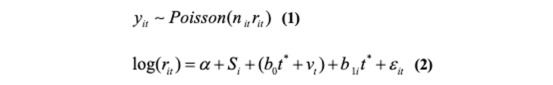



In Poisson likelihood function (equation 1), *y_it_* is the amount of online HIVST kit sales in place *i* at time *t*, *n_it_* is the number of total populations in place *i* at time *t*, and *r_it_* is the rate of online HIVST kit sales per capita in place *i* at time *t*.

In the log link regression function (equation 2) that describes the rate of online HIVST kit sales per capita, α is the overall average for the entire study period and *S_i_* is the overall spatial trend. Additionally, t* is the median time of the study period, (*b*_0_t* + *v_t_*) and *b*_1__i_t* specify the overall and local temporal trend at place *i*, respectively. ε*_it_* is the term for random Gaussian noise.

### Factors Associated With the Online Purchase Rate of HIVST Kits Per Capita

To investigate factors associated with the behavior of online purchase of HIVST kits, the ordinary least squares regression (OLSR) was performed using data in 2017 to analyze the correlation between the online purchase rate of HIVST kits per capita and 5 potential factors: (1) population density, (2) GDP per capita, (3) road density, (4) HIV/AIDS screening laboratory density, and (5) rate of newly diagnosed HIV/AIDS cases per 100,000 persons. We also used the statistical tool Geodetector [16-18] to quantify the interactive effect of every 2 factors on influencing the online purchase rate of HIVST kits per capita with a *q* statistic value. The *q* statistic value ranged between 0 and 1, and a higher *q* statistic value means stronger influencing power.

### Contribution of HIVST Through Online Purchase to HIV Diagnosis

The contribution of HIVST through online purchase to HIV diagnosis was estimated using the city-level data in 2017 with the following formula: [(Amount of HIVST kits purchased online in a city * HIV prevalence) / newly diagnosed HIV cases in a city] * 100%. Since city-level HIV prevalence was not available for this study, we used the national HIV prevalence of 0.09% instead [19]. Cities with more than 300 newly diagnosed HIV/AIDS cases in 2017 were included in the analysis.

### Data Management and Statistical Analysis

ArcGIS software (Esri) was used to calculate population density (1/km^2^), road density (km/km^2^), and HIV screening laboratory density (1/km^2^). The Bayesian hierarchical model was conducted with WinBUGS (University of Cambridge). OLSR was performed using GeoDa version 1.12.1.139 software. All geographic maps were created with ArcGIS 10.2 software.

### Ethics Approval

The study was approved by the ethics review committees of the National Center for AIDS/STD Control and Prevention, Chinese Center for Disease Control and Prevention (X131022302), and all procedures were performed in accordance with the relevant guidelines and regulations.

## Results

### Temporal Trend and Geographic Distribution of Online HIVST Kit Sales

**Figure 1:**
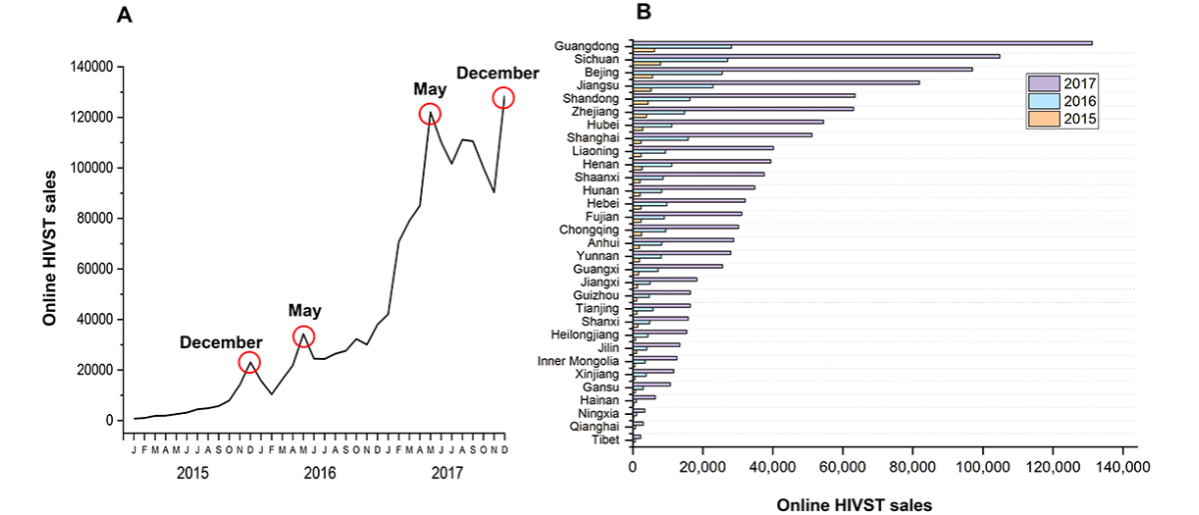
Online purchase of HIVST kits in China 2015-2017: (A) monthly online purchase amount of HIVST kits and (B) online purchase amount of HIVST kits in provinces. HIVST: HIV self-testing.

Between 2015 and 2017, a total of 1,482,773 HIVST kits were sold online. Online sales of HIVST kits showed an evident upward temporal trend and undulation over the seasonal variations, with annual peak sales occurring in May and December (Figure 1A). Among all provinces, Guangdong, Sichuan, Beijing, Jiangsu, Shandong, and Zhejiang ranked the top 6 in online purchases of HIVST kits in 2017, with sales of more than 60,000 kits in each of the provinces (Figure 1B).

**Figure 2:**
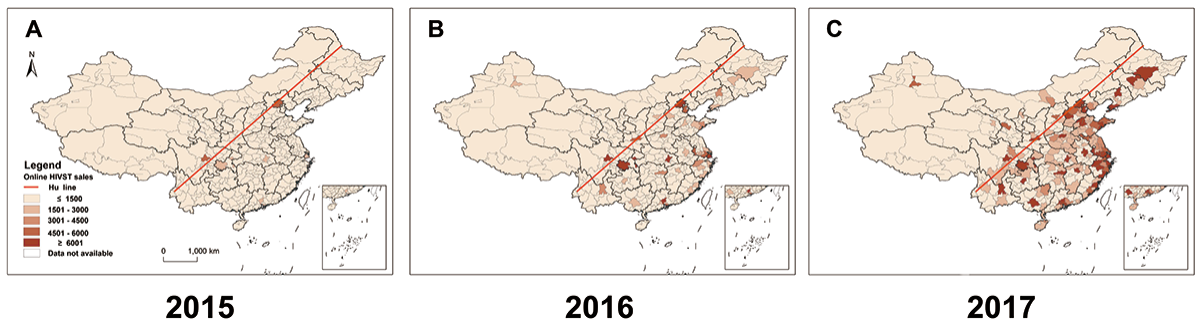
Geographic distribution of online sale amount of HIVST kits in China 2015-2017: (A) 2015 map, (B) 2016 map, and (C) 2017 map. Most HIVST kits were sold online to cities located southeast of the Heihe-Tengchong Line (as shown the red line), which marked a striking difference in the distribution of population in China. Color coded from pale orange to dark orange, and dark orange indicates high level. HIVST: HIV self-testing.

Geographic distribution analysis showed that, in 2015, only 7 major cities (Beijing, Chengdu, Chongqing, Shanghai, Shenzhen, Guangzhou, and Wuhan) purchased more than 1500 HIVST kits, and in 2017, this number had rapidly increased to 132 cities (Figure 2 A-C). Spatial distribution of online HIVST kit sales was uneven across the country (Figure 2C). If we draw a Heihe-Tengchong Line, also known as the Hu Line since 1934 [20], which marks a striking difference in the distribution of population in China, we can see that most HIVST kits were sold online to cities located southeast of the Heihe-Tengchong Line.

**Figure 3:**
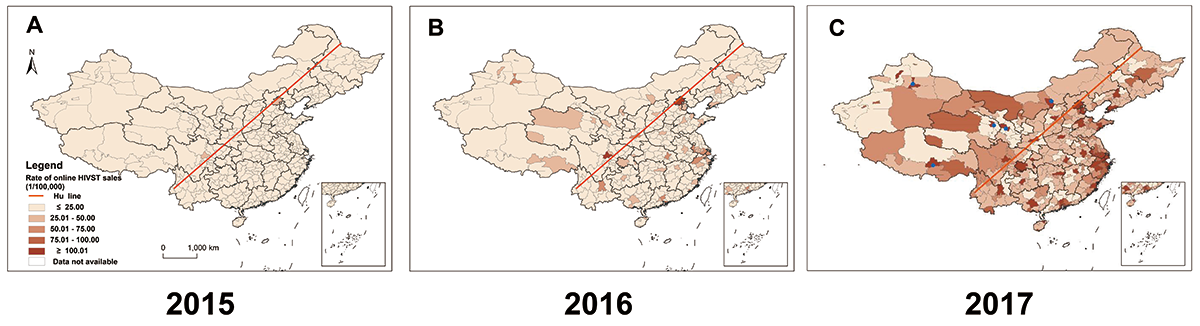
Geographic distribution of the online purchase rate of HIVST kits per capita in China 2015-2017: (A) 2015 map, (B) 2016 map, and (C) 2017 map. To exclude the impact of population density on the online sales of HIVST kits, we analyzed the geographic distribution of the online purchase rate of HIVST kits per capita. Urumchi, Lhasa, Hohhot, Sining and Lanchow (as shown in blue spots) and other cities located northwest of the Heihe-Tengchong Line showed similar online purchase rate of HIVST kits per capita in 2017 compared with cities located southwest of the Heihe-Tengchong Line (as shown the red line). Color coded from pale orange to dark orange, and dark orange indicates high level. HIVST: HIV self-testing.

To exclude the impact of population density on the online sales of HIVST kits, we analyzed the geographic distribution of the online purchase rate of HIVST kits per capita (Figure 3 A-C). Urumchi, Lhasa, Hohhot, Sining, and Lanchow (as shown in blue spots) and other cities located northwest of the Heihe-Tengchong Line showed similar online purchase rate of HIVST kits per capita in 2017 compared with cities located southwest of the Heihe-Tengchong Line (Figure 3C). These results suggested that although the online purchase amount of HIVST kits in a city appeared to be associated with the number of people in that city, the willingness to purchase HIVST kits online is uniform across the country and independent of population size.

### Spatial Distribution Characteristics of the Online Purchase Rate of HIVST Kits per 100,000 Persons

**Figure 4:**
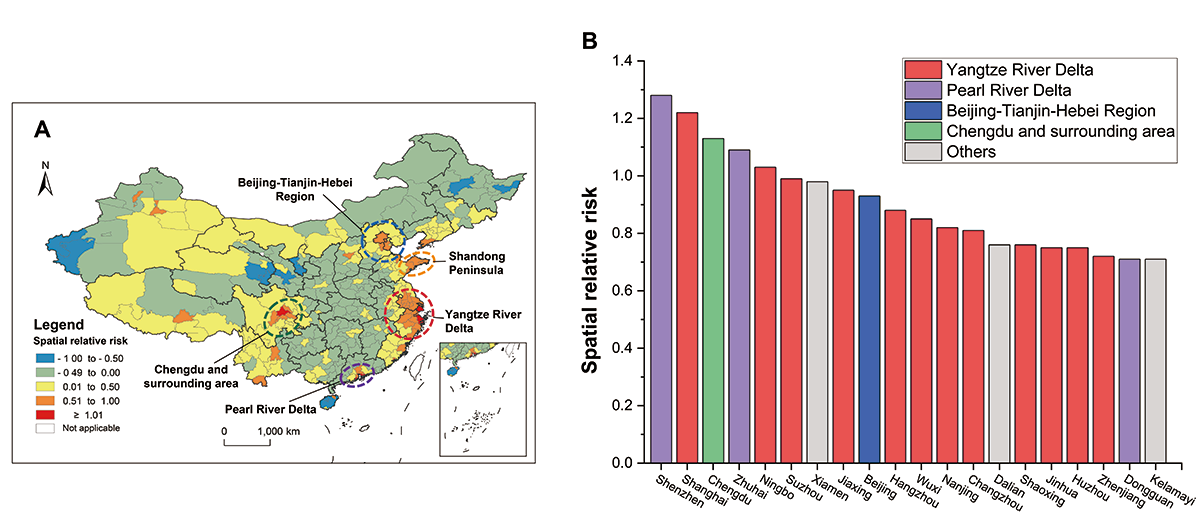
Spatial pattern of online purchase of HIV self-testing (HIVST) kits: (A) spatial pattern of online purchase preference of HIVST kits 2015-2017 and (B) the top 20 cities showed high spatial preference for online purchasing HIVST kits.

According to the results of spatial distribution analysis by the Bayesian hierarchical model, the online HIVST sales were unevenly distributed (Figure 4A). There were 5 regions that showed a comparatively higher spatial preference for purchasing HIVST kits online, including the Pearl River Delta, Yangtze River Delta, Chengdu and surrounding areas, Beijing and Tianjin areas, and Shandong Peninsula. These 5 regions also had a higher economic status than other areas in China [21], and the top 20 cities showing a high spatial preference for online purchasing of HIVST kits were mostly from these 5 regions (Figure 4B).

### Factors Associated With the Online Purchase Rate of HIVST Kits per 100,000 Persons

**Figure 5:**
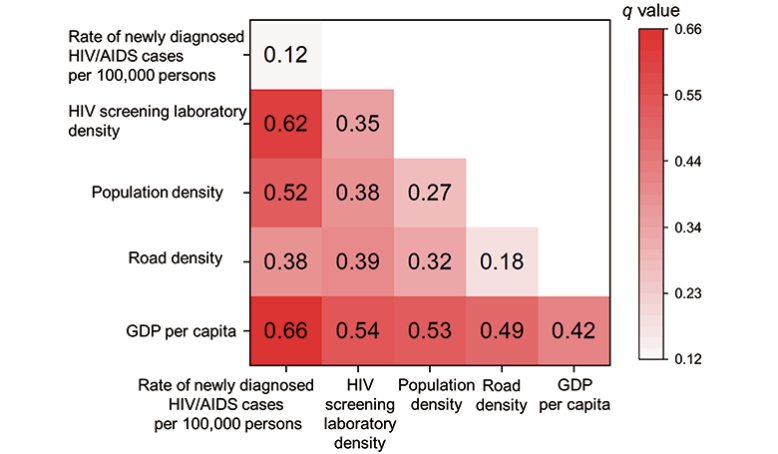
Interactive effects of potential factors on the online purchase of HIV self-testing (HIVST) kits. The darkness of color represents the value of q. GDP: gross domestic product.

We further analyzed potential factors associated with the online purchase rate of HIVST kits per 100,000 persons, including population density, GDP per capita, road density, HIV/AIDS screening laboratory density, and the rate of newly diagnosed HIV/AIDS cases per capita. OLSR analysis showed that GDP per capita and the rate of newly diagnosed HIV/AIDS cases per 100,000 persons were positively associated with the online purchase rate of HIVST kits per 100,000 persons (Table 1). Furthermore, according to the results of the Geodetector analysis (Figure 5), GDP per capita and the rate of newly diagnosed HIV/AIDS cases per 100,000 persons exerted the strongest interactive effect on the online purchase rate of HIVST kits per 100,000 persons with a *q* value of 0.66, which means that 66% heterogeneity of the rate of online HIVST kit sales per 100,000 persons can be explained by these 2 factors.

**Table 1:** Potential factors associated with the online purchase rates of HIV self-testing (HIVST) kits per 100,000 persons using ordinary least squares regression.

	Coefficient	*P* value
GDP^a^ per capita	0.001	<.001
Road density	21.65	.60
Population density	–0.004	.79
HIV screening laboratory density	4.26	.07
Rate of Newly diagnosed HIV/AIDS cases per 100,000 persons	0.99	<.001
*R* ^2^	0.48	<.001
AIC^b^	3578.22	<.001

^a^GDP: gross domestic product.

^b^AIC: Akaike information criterion.

### Contribution of Online Purchasing of HIVST Kits to the Detection of HIV Cases

We roughly estimated the contribution of online purchasing of HIVST kits to the detection of HIV-infected individuals in 2017 and found that among the top 20 cities with the highest contribution, the contribution ratio ranged from 1.28% to 3.51% (Table 2). Among the top 20 cities with comparatively higher contributions to the detection of HIV-infected individuals, 12 cities are located in the 4 major economic regions in China as shown in Figure 4A (Pearl River Delta, Yangtze River Delta, Beijing-Tianjin-Hebei Region, and Shandong Peninsula), 7 cities are provincial capitals, and 1 city is a municipality under independent planning status. When further looking at the online HIVST kit purchase amounts and the proportion of newly diagnosed HIV/AIDS cases in these 20 cities, 14 of the 20 cities with the highest contribution to the detection of HIV-infected individuals also entered the top 20 in the proportion of online HIVST kit purchase amounts, although their ranks in the proportion of newly diagnosed HIV/AIDS cases were not high. It is worth noting that among the top 20 cities with comparatively higher contributions to the detection of HIV-infected individuals, 14 cities were also identified in the top 20 cities with a high spatial preference for online purchasing HIVST kits (Figure 4B). These findings suggested that the HIV self-testing through online access contributed to the detection of HIV-infected individuals in a city; however, it was noted that the contribution ratio was not high.

**Table 2:** The top 20 cities with comparatively higher contributions to detection of HIV-infected individuals.

City	Contribution ratio (%)	Rank	Proportion of online HIVST^a^ purchase amount (%)	Rank	Proportion of newly diagnosed HIV/AIDS cases (%)	Rank	Location
Wuhan	3.51	1	3.45	5	0.63	38	Provincial capitals
Shanghai	2.69	2	4.57	4	1.11	11	Yangtze River Delta
Beijing	2.49	3	8.66	1	2.24	4	Beijing-Tianjin-Hebei Region
Guangzhou	2.38	4	4.80	3	1.30	10	Pearl River Delta
Nanjing	2.33	5	1.63	12	0.45	65	Yangtze River Delta
Hefei	2.27	6	0.95	18	0.27	96	Yangtze River Delta
Xi’an	2.24	7	2.53	8	0.73	28	Provincial capitals
Qingdao	1.99	8	0.94	19	0.31	88	Shandong Peninsula
Zhengzhou	1.94	9	1.20	15	0.40	71	Provincial capitals
Suzhou	1.89	10	1.75	11	0.60	42	Yangtze River Delta
Shenyang	1.87	11	1.84	9	0.63	39	Provincial capitals
Tianjin	1.84	12	1.47	14	0.52	60	Beijing-Tianjin-Hebei Region
Jinan	1.71	13	0.72	26	0.27	93	Provincial capitals
Hangzhou	1.56	14	1.83	10	0.76	24	Yangtze River Delta
Xiamen	1.48	15	0.62	32	0.27	95	Others
Changzhou	1.43	16	0.54	36	0.24	113	Yangtze River Delta
Wuxi	1.41	17	0.78	24	0.36	83	Yangtze River Delta
Changsha	1.29	18	1.50	13	0.75	26	Provincial capitals
Shijiazhuang	1.29	19	0.61	34	0.30	90	Beijing-Tianjin-Hebei Region
Taiyuan	1.28	20	0.48	39	0.24	112	Provincial capitals

^a^HIVST: HIV self-testing.

## Discussion

### Principal Findings

Our temporal analysis found that online HIVST kit sales in China showed an obvious undulation within a year, with significant peaks in May and December. This could be due to some special events occurring during certain time periods. In China, a week-long holiday usually begins on May 1, International Workers’ Day, during which individuals are more likely to engage in risky sexual behaviors [22,23], increasing the demand for HIVST kits. We also observed an uptick in online HIVST kit sales around World AIDS Day on December 1 [24], likely due to the high level of advocacy and campaigns to promote HIV testing during this period [25]. It is worth noting that, by the time the State Council of China released the Thirteenth Five-Year Plan (2017-2022) in January 2017 announcing that China would promote HIVST by selling HIVST kits in pharmacies and online, the online HIVST kit sales had tripled, representing a high demand and acceptance of HIV self-testing among people in need. Therefore, it is of great significance to investigate the behaviors of those who purchase HIVST kits online to better promote the HIV self-testing strategy in China.

The spatial Bayesian analyses in this study found that cities located in economically developed regions have a relatively high spatial preference for the online purchase of HIVST kits. Furthermore, regression analysis identified GDP per capita and the rate of newly diagnosed HIV/AIDS cases per 100,000 persons as 2 factors associated with the online purchase rate of HIVST kits per 100,000 persons. Therefore, our findings suggested that when HIVST was launched in China from 2015 to 2017, online purchase of HIVST was more acceptable to high-risk individuals with good financial statuses, which was consistent with our previous survey results on a small group of people who purchased HIVST online [26]. The Geodetector analysis also identified GDP per capita and the rate of newly diagnosed HIV/AIDS cases per 100,000 persons as 2 factors having the strongest interaction with the online purchase rate of HIVST kits per 100,000 persons, which echoes a previous report that income status was positively related to high-risk behaviors among men who have sex with men (MSM) in China [27].

Another interesting finding is that online HIVST sales exhibited a long-tail effect. For the traditional HIV testing services provided by medical institutions through PITC and VCT, 80% of HIV screening tests were reported to have originated from 17.3% of the laboratories [28], which showed a phenomenon of the Pareto principle (80-20 phenomenon) [29]. The Pareto principle states that approximately 80% of the effects come from 20% of the causes and is commonly used in the field of traditional place-based sales channels. In our study, when looking at the proportion of online HIVST sales at the city-level in 2017, 4 first-tier cities and 15 new first-tier cities with 16.2% of the HIV screening laboratories accounted for only 48.52% of online HIVST sales. This long-tail effect suggested that the testing delivery strategy through online purchase of HIVST kits had a more generalized effect than traditional place-based HIV testing. The long-tail effect has recently been widely used to improve public health [30], library service [31], and industrial parks [32].

In this study, we roughly estimated the contribution of online purchase of HIVST kits to detect HIV/AIDS cases in 2017 and found that the contribution rate was not high from 2015 to 2017. The highest contribution rate was only 3.5% in Wuhan city. We suspected this is because from 2015 to 2017 when HIVST was initially introduced in China and gradually implemented nationwide, self-testing was unfamiliar to people in need who had previously sought HIV testing at local hospitals, maternal centers, and public health laboratories. In addition, when estimating the contribution of online HIVST kits to newly diagnosed HIV/AIDS cases, we used national HIV prevalence (0.09%) in the general population which could underestimate the contribution ratio since HIV risk behaviors are higher among individuals who purchased HIVST kits online. A study conducted in MSM across China shows that internet-based self-test behavior was more common among first-time testers, many of whom reported a higher risk of sexual behaviors [33].

In addition, we found that the rate of newly diagnosed HIV/AIDS cases per 100,000 persons is a factor associated with online purchasing of HIVST kits, raising another important question as to whether the HIV epidemic affects the purchase of HIVST kits or the purchase of HIVST kits can indicate or predict the occurrence of the epidemic. Regardless, more research is needed to clarify these issues.

### Limitations

The rapid growth in sales of online HIVST kits from 2015 to 2017 indicates that this new HIV testing service is generally accepted by those in need. However, it is noteworthy that the causes of the increasing trend in online purchase of HIVST kits are heterogeneous. Specifically, due to the lack of data on online shopping activity, our study was unable to assess whether the increased online purchase of HIVST kits in a region is associated with the high online purchase engagement among local residents. To address this concern, future research should analyze in-depth the contribution of web development to the online purchase of HIVST kits. In addition, some information about HIVST kits sold by the 2 e-commerce platforms was missing, such as price, types of products, and retailers. These factors could also potentially impact customer behavior in online purchasing of HIVST kits.

### Conclusion

This study found that the online purchasing of HIVST kits has been generally accepted by those in need and were preferred by individuals in regions with a good economy. In addition to economic status, a higher rate of newly diagnosed HIV/AIDS cases per 100,000 persons is also associated with online purchasing of HIVST kits.

## Abbreviations

GDP: gross domestic product
HIVST: HIV self-testing
HTS: HIV testing service
MSM: men who have sex with men
OLSR: ordinary least squares regression
PITC: provider-initiated HIV testing and counseling
RDT: rapid diagnostic test
UNAIDS: Joint United Nations Program on HIV/AIDS
VCT: voluntary counseling and testing
